# New graduate registered nurses’ professional competence and the impact of preceptors’ education intervention: a quasi-experimental longitudinal intervention study

**DOI:** 10.1186/s12912-022-01133-4

**Published:** 2022-12-16

**Authors:** Kirsi Lindfors, Mervi Flinkman, Marja Kaunonen, Heini Huhtala, Eija Paavilainen

**Affiliations:** 1 Tampere University, The Department of Children and Adolescents, Helsinki University Hospital, Stenbackinkatu 9, BOX 347, 00290 Helsinki, Finland; 2 Department of the Nursing Science, University of Turku, Turku, Finland; 3 Faculty of Social Sciences, Tampere University, Pirkanmaa Hospital District General Administration, Tampere University, Tampere, Finland; 4 Faculty of Social Sciences, Tampere University, Tampere, Finland; 5 Faculty of Social Sciences, Tampere University, Etelä-Pohjanmaa Hospital District, Seinäjoki, Finland

**Keywords:** Orientation, New graduate nurse, Professional competence, Intervention study

## Abstract

**Aims and objectives:**

The aim of this quasi-experimental longitudinal intervention study was to investigate new graduate nurses’ professional competence development after preceptors’ participation in an education intervention.

**Background:**

New graduate registered nurses are expected to be competent in many areas of nursing. Expectations that are sometimes unrealistic may cause a sense of inadequacy and stress, and this may in turn prevent them from fully deploying their competencies. Competence development is related to practice environment, occupational commitment, empowerment, and work experience. Orientation or transition programs have been designed to ensure new graduate nurses’ competence, and preceptors and preceptorship could also have significant influence on their competence development.

**Design:**

A quasi-experimental longitudinal intervention study.

**Methods:**

The data was collected from October 2015 to November 2017. Participating wards were randomized into intervention and control groups. The intervention group preceptors had an eight-hour education intervention that focused on new employees’ orientation, particularly from new graduates’ point of view. Wards in the control group continued to precept as before. The Nurse Competence Scale was used for new graduates’ self-assessment at baseline and at three-month and nine-month follow-up. This study is reported in accordance with the TREND Statement Checklist.

**Results:**

The education intervention aimed at preceptors did not have impact on the intervention group NGRNs’ competence development. There were no statistically significant differences between the groups and effect size remained small.

**Conclusions:**

The preceptors’ education intervention was not effective enough to develop new graduates’ professional competence so that it would have differed from that of the graduates receiving conventional orientation at the university hospital. This study confirmed that competence development is a complex and multidimensional phenomenon and organizations should invest in new graduate registered nurses’ competence development during their early career. Preceptors’ education and development of preceptorship and transition programs are an important part of overall competence development in complex health care environments.

**Trial registration:**

Retrospectively registered

## Background

New graduate registered nurses (hereafter NGRNs) are expected to be competent in many areas of nursing after having started to work [1]. The beginning of NGRNs’ career may often be dominated by feelings of fear of making mistakes, harming a patient, the unknown future after the orientation period, and of being unable to meet expectations. Despite the feelings of fear, there are also feelings of achievement and satisfaction [2]. The NGRNs’ transition time has also been described as a process of becoming, which includes three stages: doing, being, and knowing. Through these stages, a NGRN becomes a professional who can answer questions rather than merely asking them. At the final stage, NGRNs should have reached a level of comfort and confidence with their role, responsibilities, and routines [3].

Communication skills, conflict resolution skills, organization, prioritization and time management, critical thinking skills, clinical decision-making, and stress management are crucial competences for NGRNs but are often areas that they struggle with, according to literature [1, 4]. Expectations that are sometimes unrealistic may cause sense of inadequacy and stress, and this may in turn prevent NGRNs from fully deploying their competences [5, 6]. The COVID-19 pandemic has also brought new challenges to NGRNs by decreasing graduating students’ clinical placements opportunities [7, 8], which may in turn affect NGRNs’ readiness to enter clinical practice and increase NGRNs’ feelings of fear, fatigue, and self-doubt [7, 9].

NGRNs’ competence is built through development of clinical knowledge and skills. A welcoming and safe clinical environment together with support, guidance and feedback from colleagues may enhance this development. [7, 10] Orientation or transition programs have been developed to ensure NGRNs’ competence [11]. Preceptors and preceptorship could also have a significant effect on NGRNs’ competence by supporting development of their confidence and competence during the transition period [12–14]. Stability of ward or work shift, perceived workload, wards’ positive attitude towards continuing education, and individual factors like the NGRNs themselves and professionals with whom they work with have been found to be one way to facilitate NGRNs’ transition period and give them an opportunity to progressively develop their competences [6].

NGRNs’ competence should also be seen as part of quality and patient safety [15]. Confidence, safe practice, and holistic care are factors that are achieved through nurse competence [16], and it is the moral and ethical responsibility of health care organizations to provide safe patient care by a competent workforce [17]. The more competent the graduate, the more confident he or she is about his or her skills. Awareness of one’s own limitations of knowledge and experience, courage to ask for assistance whenever needed, and awareness of nursing principles contribute towards safe practice and prevent doing harm to patients and oneself [16].

Competence has been defined in terms of functional adequacy, and the capacity to integrate knowledge, skills, attitudes, and values in specific contextual situations of practice. [18] It is a complex, relative, context-dependent, and variable concept including physical, mental, psychosocial, and social dimensions [19]. The foundation of competence development is laid already during nursing studies [20]. The level of competence develops alongside clinical experience [21], and the length of working experience is associated with better professional nurse competence [22]. NGRNs need opportunities and challenges to progressively develop their competences [5, 23]. The first years of practice provide nurses with plenty of learning opportunities, and they have the capability to absorb new knowledge and skills in the early stages of their professional career [21].

After graduation, NGRNs should possess basic knowledge of the nursing profession to act independently and to carry out vocational functions and eventually, to be ready for continuous learning. NGRNs’ ability to apply theory into practice, ethical commitment, critical thinking and problem-solving skills, and ability to work in interdisciplinary contexts have been considered the most important generic competencies [24]. The study of Brown and Crookes (2016) showed the complexity of defining the expected level of NGRNs’ competence at the point of graduation [25]. Different clinical work settings require different competences [26], and NGRNs’ abilities do not always match the expectations that nurse managers and experienced colleagues have about NGRNs’ readiness for clinical practice [5, 26].

In studies focusing on NGRNs’ professional competence, the findings highlight that professional competence is related to practice environment, occupational commitment, and empowerment [27–29], and the findings are usually based on NGRN’s self-assessed competence level [30–33]. The practice environment has been perceived more positively by NGRNs with higher competence level, and a positive practice environment, in turn, supported NGRNs’ competence, retention and job satisfaction [28]. Higher professional competence was also associated with affective occupational commitment of NGRNs, where they felt proud to be nurses [27]. Higher competence and empowerment seemed to be related to each other. Higher competence level was associated with higher moral principles, personal integrity, expertise and future-orientedness [29].

NGRNs’ self-assessed overall competence level varies in different studies measured by the Nurse Competence Scale (NCS©) from moderate to good (Visual Analogue Scale [VAS] mean 40.1–62.5) [34] to very good (VAS mean 59.5–76.7), depending on time since graduation [35]. NGRNs have assessed themselves as most competent in areas concerning individualized care, patients’ coping strategies and ethical decision-making [30, 31]. They have assessed themselves to be least competent in areas concerning professional development and nursing research [31], collaboration and evaluation of care situations [30, 32] and patient education and guidance of family-members and colleagues. [32] In the study of Lejonqvist and Kajander-Unkuri (2021), self-assessed professional competence level changed over time, and low competence levels improved to good level during six months of practice [35].

Previous studies about NGRNs’ competence have focused on the practice environment, occupational commitment, and empowerment and on NGRNs’ perceptions of self-assessed competence level during their first year after graduation [8, 27–33]. Competence-related intervention studies [36–38] have focused on more seasoned registered nurses, but studies about interventions aimed at developing NGRNs’ professional competence and preceptors’ role in competence development are lacking. Previous studies about preceptors’ training have focused on measuring NGRNs’ retention, critical thinking skills, and their stress levels [39] and perceived competence of preceptors after their education [40, 41]. The purpose of this study was to examine the development of NGRNs’ competence level after preceptors had had an eight-hour education entity about orientation and preceptorship. This study focuses on the impact of the preceptors’ education intervention on NGRNs’ professional competence by comparing the intervention and control groups’ NGRNs competence development during the nine-month follow-up period.

## Methods

### Aim

The aim of this quasi-experimental longitudinal intervention study was to investigate NGRNs’ self-assessed professional competence development over at nine-month follow-up period and to compare the NGRNs in the intervention and control group.

The research questions are:

1. How did NGRNs’ level of competence in terms of quality of action and frequency of action change during the follow-up period?
2. How did the competence level of the intervention and control group NGRNs develop during the follow-up period?
3. How did preceptors’ education intervention affect the intervention group NGRNs’ competence development?

The study hypothesis was that the education given in the intervention group preceptors would foster their knowledge about precepting and the orientation period, and this would in turn enhance NGRNs’ competence level development.

### Research design and instrument

This study was part of a larger longitudinal quasi-experimental intervention study which aimed to investigate NGRNs’ professional competence development (primary outcome), their evaluations of received orientation and organizational commitment (secondary outcomes), and the impact of preceptors’ education intervention on these outcomes. This paper focuses particularly on NGRNs’ competence level development during the nine-month follow-up period after the preceptors’ education intervention to better emphasise on the phenomenon of NGRNs’ self-assessed competence development.

A total of 194 nursing wards in one of the five university hospitals in Finland were asked to participate in this longitudinal quasi-experimental intervention study. Nearly a third (29.3%; *n* = 57) of the wards accepted the request to participate. The wards were randomized by simple random sampling into intervention group and control group. The nursing wards were divided by ward type into two categories: inpatient wards and outpatient clinics. Intensive care units, step-down units, and operation rooms (OR) were combined into a separate, a third category. The randomisation to intervention and control group was made within these ward categories using computer-generated randomisation codes and lists. After randomisation, two of the wards were merged and five of the wards declined to participate. A total of 50 (25.7%) wards participated in the study [42].

### The intervention

The intervention group preceptors (*n* = 174) were given an eight-hour face-to-face clinical education session about new employee orientation, focusing particularly on NGRNs’ viewpoint. The education intervention included lectures, discussion moments and exercises. The education intervention was based on Duchscher’s theory of transition and NGRNs’ first year [3]. Previous study findings about NGRNs’ professional competence development and preceptors’ role were important part of the education intervention [10, 12–16, 18, 27, 28, 31, 32, 42]. The content of the education intervention is shown in Table 1. The principal investigator (KL) provided the education to the preceptors, and lectures were held in the hospital lecture rooms during preceptors working days. Nurse managers selected participating preceptors. The principal investigator (KL) sent preceptors a welcoming email beforehand with an information leaflet about the upcoming education session. Education group sizes varied from 2 to 17 participants. The first objective was to enhance intervention group preceptors’ knowledge about orientation and to give preceptors and wards means to improve their precepting methods.

**Table 1:** The content of preceptors’ education intervention

Overview of the day’s content, everyone introduced themselves
Preceptor’s role and responsibilities
New graduate nurse’s first year. Also including information about new graduate nurse’s learning needs, different learning styles and exercises about learning styles, differences between generations
New graduate nurse’s critical thinking abilities and exercises to support development of critical thinking
Assessment and principles of constructive feedback, a structured checklist for orientation assessment discussions with ward manager, preceptee and preceptor
How to support new graduate nurse? Peer support and another support methods, mentoring
End of the day. Each participant was given materials about lectures and exercises above

This was the first time in this university hospital when preceptors were offered a planned education session about new employees’ orientation. All Finnish nurses are educated in the universities of applied sciences (3,5 years and 210 ECTS) and precepting is a normal part of their daily work of which they don’t have any extra compensations. Preceptors’ education about orientation and NGRNs’ challenges during their transition period has not been seen necessary and every registered nurse is obligated to act as a preceptor. In addition, preceptors don’t have any official support systems to carry out their important work as preceptors and the second objective was to foster their professional self-confidence as preceptors.

The study was registered with the ClinicalTrials.gov identifier NCT04474769 and the TREND Statement checklist has been followed [43].

### The instrument

Many different instruments have been used to measure NGRN’s competence [44]. In this study, the data was collected by using the NCS instrument by Meretoja. The NCS instrument was developed in Finland by nursing experts. A large pool of competence indicators (*n* = 1,308) was reduced by deductive content analysis to a total of 73 items, and the instrument contains two assessment scales. The NCS was later translated into many languages, and it has been used in several countries, different practice environments and nurse samples to study competence levels and the factors associated with competence and to evaluate the effects of education interventions. The NCS has shown good content validity and adequate internal consistency [34, 45]. In this study, the Cronbach’s alpha of each category varied from 0.792 to 0.925, indicating good internal consistency [46].

The NCS consists of seven competence categories: Helping Role (7 items), Teaching-Coaching (16 items), Diagnostic Functions (7 items), Managing Situations (8 items), Therapeutic Interventions (10 items), Ensuring Quality (6 items) and Work Role (19 items). The Helping Role category comprises competence areas such as supporting patients’ coping strategies, individualized and ethical care. The Teaching-Coaching category is about the education needs of patients and family members, evaluation of education outcomes, and educating colleagues. Diagnostic Functions are mainly related to emotional support of patients and their family members whereas Managing Situations is focused on rapidly changing, life-threating situations. Therapeutic Interventions are focused on critical thinking, ability to utilize research knowledge, ability to assess effectiveness of care and to co-ordinate and organize. Ensuring Quality is about identifying and promoting development of patient care areas whereas Work Role is focused on acting responsibly and autonomously, taking care of professional development of the nursing ward, and acting as a valuable team member [45].

In each of these items the competence level is self-rated by using a visual analogue scale (VAS) from 0 to 100. For descriptive purposes, the VAS is divided into four parts to represent the level of competence: VAS score 0–25 means low competence, VAS > 25–50 means quite good competence, VAS > 50–75 means good competence, and VAS > 75–100 means very good competence [34, 45].

The NCS also contains another scale (frequency of action) which measures the frequency of using different competences (items) in clinical practice on a 4-graded scale: 0 = not applicable, 1 = used very seldom, 2 = used occasionally, 3 = used very often in my work. [34, 45].

### Data collection

The data was collected by using an electronic questionnaire which included demographic data such as age, gender, ward type, previous qualifications, working experience, and the NCS instrument. The inclusion criteria were starting work as a registered nurse in his or her first workplace, upcoming orientation phase, and willingness to participate. The exclusion criterion was more than one year from graduation. NGRNs with a previous degree, e.g., licensed practical nurse, were accepted if other inclusion criteria were met. The study comprised three measurement points: at baseline, three and nine months. The baseline means the moment when the NGRN starts working in the assigned nursing ward. The three-month measurement point was chosen since by this time, the orientation period is usually over and NGRNs have been working independently for some time. The nine-month measurement point was chosen according to Duchscher’s (2008) theory of transition. At nine months, the transition crisis is beginning to ease off, NGRNs are at a relatively stable stage, and they are accepting their role as professional nurses [3].

For the control group, data collection started in October 2015. The intervention group’s wards joined in one by one after their preceptors had been educated. The data collection lasted until November 2017. Ward managers delivered participating NGRNs' email addresses to the researcher. The questionnaires were sent as follows: at the baseline to all participating NGRNs, at the three-month measurement point to all those NGRNs who had returned the questionnaire at the baseline, and the nine-month questionnaire to the NGNRs who had participated at the three-month measurement point.

### Data analysis

The sample size calculation was based on the primary outcome, professional competence, by using the NCS. The estimated standard deviation was 13. The significance level was set up to 0.05 with statistical power of 80% and difference between groups was set at 6-points. According to these assumptions, the target sample size was 75 respondents in each group [47].

The data was analyzed by using IBM SPSS Statistics (27.0) software and the p-value was set at ≤ 0.05. Descriptive statistics as frequencies, mean and standard deviation and percentage values were used to summarize the data. Mean variables were formed of each competence category and mean value of the VAS was calculated. A non-parametric test like Mann–Whitney was used to compare differences between the groups because part of the variables was not normally distributed. Even though there were variables that were not normally distributed, the Cohen d was used to determine the impact of the intervention. The frequency of action was analyzed by frequencies, percentages and by cross-tabulation. The McNemar test was used to determine the differences in frequency of action categories between baseline and nine months. The attrition analysis was conducted by comparing the intervention and control group participants at baseline, three months, and nine months by using cross-tabulation and T-test. The attrition rate was calculated. Cronbach’s alpha was used to determine the internal consistency.

### Ethical considerations

Ethical approval (98/13/03/03/2012) from the organization’s ethics committee and research permission (24/01/2014) were obtained. Each participating NGRN was given a cover letter including information about the purpose and aim of the study for them to make an independent decision on whether to participate in this study. Answering the electronic questionnaire was seen as consent to participate in the research [48]. The ward managers recruited both NGRNs and preceptors to this study. The researcher met every ward manager before the study began. At these meetings, voluntary participation was emphasized. The permit to use the Finnish NCS was given by its developer Riitta Meretoja.

## Results

A total of 114 NGNRs’ email addresses were delivered to the researcher during the follow-up. Three quarters (*n* = 95, 83%) of the NGNRs participated in the first measurement, 72 (76%) in the second measurement, and 61 (85%) in the third measurement. Most of the NGRNs were aged 20–25 years and they worked in inpatient wards. Although the participants were NGRNs and had less than one year from graduation as registered nurses, the majority had at least one year of work experience in healthcare. Nearly a quarter (23.2%) had a previous degree as licensed practical nurses. (Table 2).

**Table 2:** Participants’ background characteristics at the baseline

	Intervention group		Control group	
	*n*	%	*n*	%
Age group				
20–25 years	33	57.9	18	48.6
26–29 years	14	24.6	9	24.3
30 years and over	10	17.5	10	27.0
Gender				
Female	54	94.7	38	100.0
Male	3	5.3	0	0
Ward type				
Inpatient	34	59.6	18	47.4
Intensive and OR* units	11	19.3	14	36.8
Outpatient	12	21.1	6	15.8
Other professional qualifications				
Licensed practical nurse	11	19.3	11	28.9
Previous working experience in healthcare				
< 1 year	22	38.6	15	40.5
1–3 years	27	47.4	14	37.8
> 3 years	8	14.0	8	21.6

^*^OR Operating rooms

### The level of NGRNs’ competence during the follow-up period

The NGRNs’ level of competence in all seven categories during the follow-up was either good or very good. Throughout the follow-up period, NGRNs assessed themselves as most competent in the Helping Role category and the least competent in the Therapeutic Interventions category. During the follow-up, NGRNs’ self-assessed competence level development in each category was statistically significant (*p* ≤ 0.05). (Table 3).

**Table 3:** NGRNs’ competence level during the follow-up

	Baseline (*n* = 95)		3 months (*n* = 72)		9 months (*n* = 61)		
Competence category	Mean (SD)	α	Mean (SD)	α	Mean (SD)	α	*p*-value*
Helping role	70.4 (13.9)	0.805	76.8 (12.5)	0.825	76.0 (15.9)	0.869	0.003
Teaching-Coaching	59.6(16.5)	0.925	65.3 (17.8)	0.923	69.8 (15.2)	0.920	< 0.001
Diagnostic functions	60.6 (17.6)	0.792	65.3 (17.7)	0.848	69.0 (16.4)	0.843	< 0.001
Managing situations	57.7 (19. 9)	0.865	64.7 (18.8)	0.863	67.2 (16.9)	0.876	0.002
Therapeutic interventions	50.4 (21.6)	0.902	59.4 (19.2)	0.908	62.1 (17.8)	0.886	< 0.001
Ensuring quality	52.9 (19.9)	0.858	63.9 (17.7)	0.838	64.3 (19.5)	0.805	< 0.001
Work role	54.4 (18.7)	0.911	63.2 (15.1)	0.880	67.2 (15.8)	0.882	< 0.001
Overall competence level	58.0 (15.5)		65.5 (14.4)		67.6 (14.3)		< 0.001

^*^ = Difference between 0 and 9 months, *α*  Cronbach alpha, *p*-value ≤ 0.05, *SD*  Standard deviation, *NGRN*  New graduate registered nurse

At item-level analysis, NGRNs assessed themselves during the follow-up at very good competence level (VAS > 75) on several items. These items related to supporting patient or patient care planning or decision-making guided by ethical values (Helping Role), patient education or maintaining and improving own professional skills (Teaching-Coaching), analyzing patient’s well-being (Diagnostic Functions), NGRN’s ability to plan own activities flexibly according to clinical situations (Therapeutic Interventions), commitment to organization’s care philosophy (Ensuring Quality), professional identity or utilizing information technology (Work Role).

Low competence level (VAS score 0–25) at the baseline was reported concerning items related to Work Role (mentoring new employees or nursing students, acting as an expert in care teams, guiding staff members and leading situations), Therapeutic Interventions (developing guidelines and clinical paths), Teaching-Coaching (developing orientation programs) and Ensuring Quality (making research and development proposals). However, the NGRNs’ competence level developed quickly, and at three-months follow-up, all except one item (Mentoring novices and advanced beginners, VAS mean 23.1, SD 27.9) were at quite good competence level (VAS > 25 to 50).

The participants were also asked to evaluate the frequency with which they used each competence by using a 4-graded scale. The frequency of actions used occasionally or very often is seen on the Table 4.

**Table 4:** Frequency of action use as occasionally or very often

	Baseline		3 months		9 months		
Competence category	*n*	%	n	%	*n*	%	*p*-value*
Helping role	89	93.7	70	97.2	58	95.1	0.500
Teaching-coaching	61	64.2	48	66.6	45	73.8	0.064
Diagnostic functions	71	74.8	51	70.8	49	80.4	0.057
Managing situations	65	68.4	53	73.6	44	72.1	**0.035**
Therapeutic interventions	47	49.5	38	52.7	36	59.0	**0.031**
Ensuring quality	61	64.3	49	68.0	49	80.3	**0.003**
Work role	52	54.8	43	59.8	43	70.5	**0.002**

^*^Difference between baseline and nine-months McNemar, *p*-value ≤ 0.05, statistically significant values are bolded

The NGRNs reported using the most frequently competencies in the Helping Role category and Diagnostic Functions. The least frequently used competencies were in the Therapeutic Intervention category. When comparing the frequency differences between the baseline and nine-month follow-up, statistically significant differences were seen in the categories Managing Situations, Therapeutic Interventions, Ensuring Quality and Work Role (Table 4). An item level analysis revealed that at the items where NGRNs assessed themselves to be at low competence level (VAS < 25, at the baseline), the frequency of action was also low. On the items where NGRNs assessed themselves to be at very good competence level (VAS ≥ 75, at the baseline), the frequency of action was high.

### Competence level development within the intervention and control groups during the follow-up period

When analyzing the competence development between the intervention and control group, only those participants (*n* = 61; intervention group *n* = 36 and control group *n* = 25) who participated in all three measurements were included. NGRNs in both groups assessed themselves as most competent in the Helping Role throughout the follow-up period while the lowest competence was reported in Therapeutic Interventions. NGRNs in both groups assessed their overall competence level to be at good level (VAS > 50–75) throughout the follow-up period. (Table 5).

**Table 5:** Competence level, quality of actions

	Baseline			3 months			9 months			
	Intervention group	Control group		Intervention group	Control group		Intervention group	Control group		
Competence category	Mean (SD)	Mean (SD)	*p*-value	Mean (SD)	Mean (SD)	*p*-value	Mean (SD)	Mean (SD)	*p*-value	d*
Helping role	69.9 (14.4)	70.6 (15.0)	0.651	76.6 (13.5)	78.4 (12.0)	0.633	74.9 (16.6)	77.5 (14.9)	0.482	-.174
Teaching-coaching	60.5 (15.7)	55.5 (18.4)	0.339	64.5 (19.8)	67.6 (17.7)	0.614	70.4 (15.7)	68.93 (14.7)	0.821	.097
Diagnostic functions	63.1 (17.7)	56.3 (20.7)	0.195	64.2 (20.4)	66.4 (17.4)	0.776	71.1 (17.4)	66.1 (14.7)	0.150	.310
Managing situations	63.4 (20.1)	50.13 (21.3)	**0.024**	65.6 (20.2)	65.6 (20.7)	1.000	68.5 (18.5)	65.4 (14.6)	0.457	.181
Therapeutic interventions	54.3 (25.6)	43.9 (17.3)	0.196	59.3 (22.6)	62.1 (15.9)	0.686	60.4 (20.2)	64.4 (14.2)	0.438	-.223
Ensuring quality	55.9 (19.8)	47.3 (19.4)	0.211	65.0 (19.1)	62.3 (16.9)	0.601	65.8 (19.3)	62.4 (20.0)	0.728	.172
Work role	56.6 (20.3)	51.9 (18.9)	0.374	64.5 (17.0)	62.5 (15.6)	0.727	66.5 (18.8)	68.2 (10.9)	0.645	-.102
Overall competence level	60.5 (16.5)	54.2 (15.5)	0.180	65.7 (16.5)	66.4 (14.5)	0.755	67.7 (15.8)	67.6 (12.2)	0.937	.007

Mann–Whitney, *p*-value ≤ 0.05, statistically significant values are bolded, *Cohen d at nine-month follow-up, *SD*   Standard deviation

Comparing competence at the nine-month follow-up, competence development was statistically significant (*p* ≤ 0.05) in six categories (Helping Role from mean 69.9 to mean 74.9, Teaching-Coaching from 60.5 to 70.4, Diagnostic Functions from 63.1 to 71.1, Therapeutic Interventions from 54.3 to 60.4, Ensuring Quality from 55.9 to 65.8, Work Role from 56.6 to 66.5) within the intervention group. In the control group, competence development was statistically significant (*p* ≤ 0.05) in all categories (Helping Role from 70.6 to 77.6, Teaching-Coaching from 55.5 to 68.9, Diagnostic Functions from 56.3 to 66.1, Managing Situations from 50.1 to 65.4, Therapeutic Interventions from 43.9 to 64.4, Ensuring Quality from 47.3 to 62.4, Work Role from 51.9 to 68.2). At item level, the most item level competence development occurred in the Ensuring Quality category in both groups.

When assessing the frequency of action, the most frequently used category in both groups was the Helping Role category (Table 6). In the Diagnostic functions, Managing situations and Therapeutic Interventions categories, the intervention group NGRNs’ frequency of action was lower than that of the control group, and it remained lower throughout the follow-up period. On items related to mentoring student nurses and new colleagues (Work Role), acting as an expert in caring team (Work Role), developing caring processes (Therapeutic Interventions), making proposals about further development and research (Ensuring Quality) and developing new nurses’ orientation (Teaching and Coaching), frequency of action remained low throughout the follow-up period in both groups.

**Table 6:** Frequency of action, occasionally or very often (%)

	Baseline					3 months					9 months				
	Intervention group		Control group			Intervention group		Control group			Intervention group		Control group		
Competence category	*n*	%	*n*	%	*p*-value	*n*	%	n	%	*p*-value	*n*	%	*n*	%	*p*-value
Helping role	35	97.2	22	88.0	0.857	34	94.5	21	84.0	0.728	34	94.4	24	96.0	0.083
Teaching-coaching	21	58.3	14	56.0	0.987	20	55.5	17	68.0	0.386	26	72.3	19	76.0	0.278
Diagnostic functions	22	61.1	21	84.0	**0.024**	24	66.6	17	68.0	0.763	26	72.2	23	92.0	0.068
Managing situations	21	58.3	16	64.7	0.640	24	66.7	17	68.0	0.346	24	66.7	20	80.0	0.261
Therapeutic interventions	13	36.1	14	56.0	0.145	17	47.2	15	60.0	0.615	18	50.0	18	72.0	0.114
Ensuring quality	18	50.0	18	72.0	0.112	23	63.9	15	60.0	0.436	26	77.8	21	84.0	0.155
Work Role	16	44.4	15	60.0	0.300	21	58.3	12	48.0	0.264	23	63.9	20	80.0	0.276
Overall frequency of actions	20	55.6	19	76.0	0.169	25	69.4	16	64.0	0.570	28	77.8	22	88.0	0.324

Mann–Whitney, *p*-value ≤ 0.05, statistically significant values are bolded

#### The impact of the intervention

When comparing the competence development between the intervention and the control group and assessing the possible impact of the intervention no statistically significant differences could be observed between the groups in any of the seven competence categories at end of the follow-up and the effect size (Cohen d) remained small.

When comparing the frequency of action, the control group’s frequency of action was in general higher in all but one category throughout the nine-month follow-up period. There were no statistically significant differences between the groups in frequency of action, either (Table 6).

#### Discussion

The aim of this quasi-experimental longitudinal intervention study was to investigate NGRNs’ professional competence development and the impact of preceptors’ education intervention on it. In this study, NGRNs in both groups showed quite good or good competence level at the baseline, and their competence level improved during the follow-up period. At nine-month follow-up, the NGRNs’ self-assessed competence level was to some extent even higher than experienced nurses’ self-assessed competence level in previous studies [49–52]. However, there were no statistically significant differences between the groups and effect size remained small. In the light of these findings, the education intervention aimed at preceptors didn’t have impact on the intervention group NGRNs’ competence development.

The findings of this study revealed that NGRNs evaluated their competence level in the category Helping Role as good at the baseline, and at nine-month follow-up, the competence level was very good. The lowest competences were seen in the categories Therapeutic Interventions and Ensuring Quality. Similar findings can be seen in previous studies about NGRNs [30, 31, 49] and in studies concerning experienced registered nurses [49–53]. Even though Therapeutic Interventions and Ensuring Quality remained the lowest competence categories throughout the follow-up period, the competence in these categories was at good level. The findings of this study suggest that NGRNs feel themselves the most competent in areas concerning individualized care, ethical decision-making and helping patients to cope. These competences align with the competences that are required from graduate nurses at the point of registration [24] and from this point of view, it seems that nursing education has been successful. A similar conclusion was made by Kajander-Unkuri et al. (2014) in their study about nursing students’ competence during their final clinical placement [54].

Flexible decision-making, utilizing new knowledge on patient care, acting as a consultant to other team members and identifying and promoting patient care development initiatives seem to be competence areas where NGRNs possessed the lowest competences. These findings are quite natural when we know that NGRNs struggle with organization, prioritization, time management and clinical decision-making and critical thinking skills. These skills are crucial but difficult to implement at the beginning of one’s career [1]. Over time, as NGRNs become familiar with routines and assignments, they can get a grip on more complex competences [55]. Duchscher (2008) has named the final stage of NGRNs’ transition the “knowing” stage. This stage comprises the last nine to twelve months of NGRNs’ first year. At this stage, NGRNs have reached “a relatively stable level of comfort and confidence with their roles, responsibilities, and routines”. (3, p. 447) The statistically significant development of all competence categories can be interpreted as a sign of this kind of development. It is possible that at the nine-month follow-up, the NGRNs in this study may have reached a balance between their responsibilities and duties and were able to take possession of different competence areas more broadly. Their journey towards nursing professionals was well on its way.

Professional competence develops alongside working experience [21, 22] and it requires a versatile and positive working environment, the ability to utilize theoretical knowledge, as well as personal motivation and curiosity [6]. In this study, competence level and frequency of action were aligned with each other. This was seen when observing the findings at both category level and item level. When the frequency of action was high, the competence level was also high, and vice versa. The findings are in line with previous studies [30, 31, 45] and indicate that competence development is dependent on NGRNs’ opportunities to practice their vocational functions and to gain expertise [5, 23]. Interestingly, when comparing NGRNs in the intervention and control group, even though the control group’s frequency of action remained higher throughout the follow-up especially in Diagnostic functions, Managing situations and Ensuring quality categories, their competence level remained alike with the intervention group’s competence level. This finding is intriguing, and to some extent, it challenges previous study results where higher frequency of action predicted higher competence level [30, 31, 45]. Without additional knowledge, it is difficult to make further conclusions about the reasons why competence level and frequency of action in the control group did not align each other. This finding may reflect something about NGRNs’ ability to make self-assessments and their perceived confidence level. NGRNs do not necessarily know what they are expected to know even though there are not expected to be competent or ready for work [26, 30]. NGRNs may feel the pressure of high expectations and feelings of insecurity [2, 4] and this, in turn, may affect their self-evaluation, making them too critical.

Even though the education intervention didn’t succeed, the study provided important information about NGRNs’ competence development during their first nine months when they go through various development stages adjusting their personal and professional roles [3] and when they may feel the most vulnerable. It also provided insight into NGRNs’ competence areas where they need more practice and support to become competent professionals. With these results we can develop both transition to practice programs and support systems to take care of our newcomers and in the long run, commit them to the organization as well as the profession.

## Conclusions

NGRNs evaluated their overall professional competence level as good, and the competence level improved over the nine-month follow-up period. However, no statistically significant differences could be observed between the groups in any of the seven competence categories at end of the follow-up. Although the original objective of the intervention was not successful, NGRNs’ competence level evolved towards more complex competences during the follow-up period. Ability to utilize research knowledge, ability to assess effectiveness of care, co-ordinate and organize, delegate, and prioritize are competences which develop alongside experience, as was seen in this study. NGRNs should practice these competences from the very beginning of their nursing career to become competent professional who possess abilities to develop nursing care. This is vital for nursing and its future. We should not abandon the thought of preceptors’ education intervention, either. In the complex health care environment where organizations struggle with shortage of nurses and the COVID-19 pandemic is diminishing NGRNs’ readiness for practice, we need different kinds of interventions to develop NGRNs’ professional competence during their transition period. With different competence development initiatives, such as supportive orientation and preceptors’ education, we can foster NGRNs’ professional identity and commitment to healthcare.

### Limitations

The data collection from one university hospital in Finland made this study geographically limited by reducing the representativeness of the result. The sample size also remained small despite the relatively long study period (two years) and the numerous reminders send to ward managers and participants. Even though the original target sample was not attained, the data collection had to be stopped after two years. Some of the wards were merging and this would have affected the study significantly. The follow-up period was relatively long, nine months, containing three individual measurement points. Questions about professional competence may feel difficult to answer and the questionnaires were long, and this may have caused reluctance to participate in the survey. The small sample size may be the reason why no significant relationships or differences could be indicated. [56] The attrition rate of this follow-up study was moderate, 35.8% (*n* = 34). Twenty-one participants (36.8%) from the intervention group and thirteen (34.2%) from the control group dropped out during the nine-month follow-up period. Only one male from the intervention group remained in study throughout the follow-up period. There were no differences in age, working experience, ward type or other professional qualifications between the participants who remained in the study and those who were lost to attrition. Despite these limitations, the results provide an interesting viewpoint to NGRNs’ first year of practice and their professional competence development.

## Data Availability

The datasets generated and/or analysed during the current study are not publicly available due to a reason that data is a part of unpublished dissertation and data is in Finnish but are available from the corresponding author on reasonable request.

## Abbreviations

NGRN: New graduate registered nurse
OR: Operating rooms
NCS: Nurse Competence Scale
VAS: Visual Analogue Scale
